# Supplementary material for: Polyploidy in the adult Drosophila brain
Source: eLife. 2020 Aug 25;9:e54385. doi: 10.7554/eLife.54385 (PMC7447450; doi:10.7554/eLife.54385)
Supplement: Figure 5—figure supplement 2—source data 1. [file elife-54385-fig5-figsupp2-data1.docx]

**Figure 5-figure supplement 2-source data 1 . List of Comparisons and references for validation of RNAseq dataset using published data.**

Genes used for analysis graphed in Figure 5-Figure Supplement 2 are tabulated here with references.

| **Gene(s)** | **Comparison** | **Reference(s)** |
| --- | --- | --- |
| ubx , abd-a, abd-b | Enriched in VNC vs CB | [(Estacio-Gómez et al., 2013)](http://sciwheel.com/work/citation?ids=7099684&pre=&suf=&sa=0) |
| antp | Enriched in VNC vs CB | [(Kuert et al., 2014)](http://sciwheel.com/work/citation?ids=656000&pre=&suf=&sa=0) |
| dilp7 | Enriched in VNC vs CB | [(Nässel et al., 2008)](http://sciwheel.com/work/citation?ids=186524&pre=&suf=&sa=0) |
| Ilp2, ilp3, ilp5 | Enriched in CB vs VNC | [(Cao et al., 2014)](http://sciwheel.com/work/citation?ids=6859492&pre=&suf=&sa=0) |
| dh44 | Enriched in CB vs VNC | [(Dus et al., 2015)](http://sciwheel.com/work/citation?ids=1383608&pre=&suf=&sa=0) |
| NPF | Enriched in CB vs VNC | [(Shao et al., 2017)](http://sciwheel.com/work/citation?ids=9128925&pre=&suf=&sa=0) |
| crz | Enriched in CB vs VNC | [(Lee et al., 2008)](http://sciwheel.com/work/citation?ids=7246913&pre=&suf=&sa=0) |
| NPF, poxn, pros, imp | Enriched in CB vs OL | [(Davie et al., 2018)](http://sciwheel.com/work/citation?ids=5473689&pre=&suf=&sa=0) |
| ilp2, ilp3 | Enriched in CB vs OL | [(Cao et al., 2014)](http://sciwheel.com/work/citation?ids=6859492&pre=&suf=&sa=0) |
| ort | Enriched in OL vs CB | [(Hong et al., 2006)](http://sciwheel.com/work/citation?ids=463885&pre=&suf=&sa=0) |
| scro | Enriched in OL vs CB | [(Davie et al., 2018)](http://sciwheel.com/work/citation?ids=5473689&pre=&suf=&sa=0) |
| vsx2 | Enriched in OL vs CB | [(Erclik et al., 2008)](http://sciwheel.com/work/citation?ids=483532&pre=&suf=&sa=0) |
| fusl | Enriched in OL vs CB | [(Long et al., 2008)](http://sciwheel.com/work/citation?ids=53624&pre=&suf=&sa=0) |
| erm | Enriched in OL vs CB | [(Peng et al., 2018)](http://sciwheel.com/work/citation?ids=5003002&pre=&suf=&sa=0) |
| rh7 | Enriched in OL vs CB | [(Kistenpfennig et al., 2017)](http://sciwheel.com/work/citation?ids=9128934&pre=&suf=&sa=0) |
| soxN | Enriched in OL vs CB | [(Schilling et al., 2019)](http://sciwheel.com/work/citation?ids=7657940&pre=&suf=&sa=0) |
| vsx1 | Enriched in OL vs CB | [(Davie et al., 2018)](http://sciwheel.com/work/citation?ids=5473689&pre=&suf=&sa=0) |
| rad50,CG6465,swim, ND-ACP,ND-B18, nrv2 | Differential expression in 21d vs 2d | [(McCarroll et al., 2004)](http://sciwheel.com/work/citation?ids=1368897&pre=&suf=&sa=0) |
| mt:lrRNA, TM4SF, sta | Differential expression in 21d vs 2d | [(Davie et al., 2018)](http://sciwheel.com/work/citation?ids=5473689&pre=&suf=&sa=0) |
| Firl, Mpc1, Mpcp2, NP15.6, CG11876, CG11752, ATPsyngamma, blw, mt:ND4, mt:ATPase8, kdn, ATPsynC, ATPsynB, ATPsynD | Genes involved in oxidative phosphorylation that decline with age (Downregulated at 21d compared to 2d) | [(Davie et al., 2018)](http://sciwheel.com/work/citation?ids=5473689&pre=&suf=&sa=0) |
| Mmp1, p38a, p38c, Traf4 | Injury/Stress response (Enriched in 21d vs 2d) | [(Purice et al., 2017)](http://sciwheel.com/work/citation?ids=4121001&pre=&suf=&sa=0) |
| roX1, roX2, sxe2, fs(1)Yb, Ndc80, FucTC, | Enriched in male vs female | [(Catalán et al., 2012; Chang et al., 2011)](http://sciwheel.com/work/citation?ids=2107303,965333&pre=&pre=&suf=&suf=&sa=0,0) |
| Sxl, fru, Yp2, Yp1, Yp3, fit | Enriched in female vs male | [(Chang et al., 2011)](http://sciwheel.com/work/citation?ids=965333&pre=&suf=&sa=0) |

-

[**Bibliography**](http://sciwheel.com/work/bibliography)

[Cao, J., Ni, J., Ma, W., Shiu, V., Milla, L.A., Park, S., Spletter, M.L., Tang, S., Zhang, J., Wei, X., Kim, S.K., Scott, M.P., 2014. Insight into insulin secretion from transcriptome and genetic analysis of insulin-producing cells of Drosophila. Genetics 197, 175–192. doi:10.1534/genetics.113.160663](http://sciwheel.com/work/bibliography/6859492)

[Catalán, A., Hutter, S., Parsch, J., 2012. Population and sex differences in Drosophila melanogaster brain gene expression. BMC Genomics 13, 654. doi:10.1186/1471-2164-13-654](http://sciwheel.com/work/bibliography/2107303)

[Chang, P.L., Dunham, J.P., Nuzhdin, S.V., Arbeitman, M.N., 2011. Somatic sex-specific transcriptome differences in Drosophila revealed by whole transcriptome sequencing. BMC Genomics 12, 364. doi:10.1186/1471-2164-12-364](http://sciwheel.com/work/bibliography/965333)

[Davie, K., Janssens, J., Koldere, D., De Waegeneer, M., Pech, U., Kreft, Ł., Aibar, S., Makhzami, S., Christiaens, V., Bravo González-Blas, C., Poovathingal, S., Hulselmans, G., Spanier, K.I., Moerman, T., Vanspauwen, B., Geurs, S., Voet, T., Lammertyn, J., Thienpont, B., Liu, S., Aerts, S., 2018. A Single-Cell Transcriptome Atlas of the Aging Drosophila Brain. Cell 174, 982-998.e20. doi:10.1016/j.cell.2018.05.057](http://sciwheel.com/work/bibliography/5473689)

[Dus, M., Lai, J.S.-Y., Gunapala, K.M., Min, S., Tayler, T.D., Hergarden, A.C., Geraud, E., Joseph, C.M., Suh, G.S.B., 2015. Nutrient Sensor in the Brain Directs the Action of the Brain-Gut Axis in Drosophila. Neuron 87, 139–151. doi:10.1016/j.neuron.2015.05.032](http://sciwheel.com/work/bibliography/1383608)

[Erclik, T., Hartenstein, V., Lipshitz, H.D., McInnes, R.R., 2008. Conserved role of the Vsx genes supports a monophyletic origin for bilaterian visual systems. Curr. Biol. 18, 1278–1287. doi:10.1016/j.cub.2008.07.076](http://sciwheel.com/work/bibliography/483532)

[Estacio-Gómez, A., Moris-Sanz, M., Schäfer, A.-K., Perea, D., Herrero, P., Díaz-Benjumea, F.J., 2013. Bithorax-complex genes sculpt the pattern of leucokinergic neurons in the Drosophila central nervous system. Development 140, 2139–2148. doi:10.1242/dev.090423](http://sciwheel.com/work/bibliography/7099684)

[Hong, S.-T., Bang, S., Paik, D., Kang, J., Hwang, S., Jeon, K., Chun, B., Hyun, S., Lee, Y., Kim, J., 2006. Histamine and its receptors modulate temperature-preference behaviors in Drosophila. J. Neurosci. 26, 7245–7256. doi:10.1523/JNEUROSCI.5426-05.2006](http://sciwheel.com/work/bibliography/463885)

[Kistenpfennig, C., Grebler, R., Ogueta, M., Hermann-Luibl, C., Schlichting, M., Stanewsky, R., Senthilan, P.R., Helfrich-Förster, C., 2017. A New Rhodopsin Influences Light-dependent Daily Activity Patterns of Fruit Flies. J. Biol. Rhythms 32, 406–422. doi:10.1177/0748730417721826](http://sciwheel.com/work/bibliography/9128934)

[Kuert, P.A., Hartenstein, V., Bello, B.C., Lovick, J.K., Reichert, H., 2014. Neuroblast lineage identification and lineage-specific Hox gene action during postembryonic development of the subesophageal ganglion in the Drosophila central brain. Dev. Biol. 390, 102–115. doi:10.1016/j.ydbio.2014.03.021](http://sciwheel.com/work/bibliography/656000)

[Lee, G., Kim, K.-M., Kikuno, K., Wang, Z., Choi, Y.-J., Park, J.H., 2008. Developmental regulation and functions of the expression of the neuropeptide corazonin in Drosophila melanogaster. Cell Tissue Res. 331, 659–673. doi:10.1007/s00441-007-0549-5](http://sciwheel.com/work/bibliography/7246913)

[Long, A.A., Kim, E., Leung, H.-T., Woodruff, E., An, L., Doerge, R.W., Pak, W.L., Broadie, K., 2008. Presynaptic calcium channel localization and calcium-dependent synaptic vesicle exocytosis regulated by the Fuseless protein. J. Neurosci. 28, 3668–3682. doi:10.1523/JNEUROSCI.5553-07.2008](http://sciwheel.com/work/bibliography/53624)

[McCarroll, S.A., Murphy, C.T., Zou, S., Pletcher, S.D., Chin, C.-S., Jan, Y.N., Kenyon, C., Bargmann, C.I., Li, H., 2004. Comparing genomic expression patterns across species identifies shared transcriptional profile in aging. Nat. Genet. 36, 197–204. doi:10.1038/ng1291](http://sciwheel.com/work/bibliography/1368897)

[Nässel, D.R., Enell, L.E., Santos, J.G., Wegener, C., Johard, H.A.D., 2008. A large population of diverse neurons in the Drosophila central nervous system expresses short neuropeptide F, suggesting multiple distributed peptide functions. BMC Neurosci. 9, 90. doi:10.1186/1471-2202-9-90](http://sciwheel.com/work/bibliography/186524)

[Peng, J., Santiago, I.J., Ahn, C., Gur, B., Tsui, C.K., Su, Z., Xu, C., Karakhanyan, A., Silies, M., Pecot, M.Y., 2018. Drosophila Fezf coordinates laminar-specific connectivity through cell-intrinsic and cell-extrinsic mechanisms. elife 7. doi:10.7554/eLife.33962](http://sciwheel.com/work/bibliography/5003002)

[Purice, M.D., Ray, A., Münzel, E.J., Pope, B.J., Park, D.J., Speese, S.D., Logan, M.A., 2017. A novel Drosophila injury model reveals severed axons are cleared through a Draper/MMP-1 signaling cascade. elife 6. doi:10.7554/eLife.23611](http://sciwheel.com/work/bibliography/4121001)

[Schilling, T., Ali, A.H., Leonhardt, A., Borst, A., Pujol-Martí, J., 2019. Transcriptional control of morphological properties of direction-selective T4/T5 neurons in Drosophila. Development 146. doi:10.1242/dev.169763](http://sciwheel.com/work/bibliography/7657940)

[Shao, L., Saver, M., Chung, P., Ren, Q., Lee, T., Kent, C.F., Heberlein, U., 2017. Dissection of the Drosophila neuropeptide F circuit using a high-throughput two-choice assay. Proc Natl Acad Sci USA 114, E8091–E8099. doi:10.1073/pnas.1710552114](http://sciwheel.com/work/bibliography/9128925)
