# Supplementary material for: Polyploidy in the adult Drosophila brain
Source: eLife. 2020 Aug 25;9:e54385. doi: 10.7554/eLife.54385 (PMC7447450; doi:10.7554/eLife.54385)
Supplement: Supplementary file 1. [file elife-54385-supp1.docx]

##### **Supplemental Table 1 List of antibodies and stains used**

| **Antibody/Stain** | **Concentration** | **Source** |
| --- | --- | --- |
| rat anti-ELAV | 1 : 100 | DHSB |
| mouse anti-pH2AV | 1 : 100 | DHSB |
| mouse anti-Repo | 1 : 100 | DHSB |
| mouse anti-Lamin | 1:100 | DHSB |
| rat-anti ELAV | 1:100 | DHSB |
| DAPI | 1 : 1000 | Sigma-Aldrich |
| Alexa Fluor 568 goat anti-mouse | 1 : 1000 | ThermoFisher |
| Alexa Fluor 568 goat anti-rat | 1 : 1000 | ThermoFisher |
| Alexa Fluor 488 goat anti-mouse | 1 : 1000 | ThermoFisher |
| Alexa Fluor 488 goat anti-rat | 1 : 1000 | ThermoFisher |
| Dye-cycle violet | 2µL in 1000µL | ThermoFisher |
| Sytox Green | 2µL in 1000µL | ThermoFisher |
| Propidium Iodide | 2.25µL in 1000µL | Sigma-Aldrich |
