# Supplementary material for: Polyploidy in the adult Drosophila brain
Source: eLife. 2020 Aug 25;9:e54385. doi: 10.7554/eLife.54385 (PMC7447450; doi:10.7554/eLife.54385)
Supplement: Supplementary file 2. [file elife-54385-supp2.docx]

**Supplemental Table 2. List of Fly Stocks**

| **Stock** | **Comment** | **Source** |
| --- | --- | --- |
| *w^1118^* | isogenic | BDSC 5905 |
| *Canton-S* | WT | O. Shafer lab |
| *Oregon-R* | WT | C. Collins lab |
| *Drosophila americana* |  | P. Wittkopp lab |
| *Drosophila mauritiana* |  | P. Wittkopp lab |
| *glass^60J^* | Mutant for glass, adult flies show disorganised ommatidial structures and fail to exhibit phototaxis | O. Shafer lab |
| *w;nSyb-GAL4/Cyo* | pan-neuronal | M. Dus lab |
| *w;+;nSyb-GAL4* | pan-neuronal | M. Dus lab |
| *w;UAS-nGFP;Repo-GAL4, tubulin GAL80TS* | pan-glial | Buttitta lab stocks |
| *w;UAS-nGFP* |  | Buttitta lab stocks |
| *w;+;UAS-nGFP* |  | Buttitta lab stocks |
| *w;Moody-GAL4* | Sub-perineurial glia | C. Collins lab via Klambt Lab |
| *y,w;mz19-mCD8::GFP* | Antennal lobe projection neuron | BDSC 23300 |
| *w1118;ELAV-GAL4,UAS-nGFP* | pan-neuronal | BDSC 49226 |
| *y,w;breathless-GAL4* | Trachea | DGRC Kyoto |
| *w-;GAD1-GAL4/SM6* | GABAergic | O. Shafer lab |
| *w-;OK371-GAL4,UASn-GFP* | Glutamatergic | Buttitta lab stocks |
| *w;ChaT-GAL4* | Cholinergic | O. Shafer lab |
| *w^1118^;+;GMR-12C11-GAL4* | Tm3a | BDSC 76324 |
| *w^1118^;+;GMR-42H01-GAL4* | Dm9 | BDSC 48150 |
| *w^1118^;+;GMR-23G11-GAL4* | Dm4 | BDSC 49043 |
| *w^1118^;+;GMR-30B06-GAL4* | Dm10 | BDSC 47529 |
| *w^1118^;+;GMR-26H07-GAL4* | Dm2 | BDSC 49204 |
| *y;w;NP3233-GAL4/Cyo* | Astrocyte-like | DGRC Kyoto |
| *y;w;NP2222-GAL4/Cyo* | Cortex glia | DGRC Kyoto |
| *w;mz97-GAL4* | Wrapping glia | C. Collins lab via Klambt Lab |
| *y,w,UASmCD8::RFP,LexAop2-mCD8::GFP; CoinFLP-LexA::GAD.GAL4* | CoinFLP | BDSC 59270 and 59271 |
| *y,w,hs-FLP;LexAop-nRFP; UAS-nGFP* | hs-FLP used with with CoinFLP nuclear GFP and RFP | Buttitta lab stocks |
| *ey-FLP* | ey-FLP | BDSC 5576 |
| *y,sev,w;UAS-cdc6^RNAi^* | cdc6^KD^ | BDSC 55734 |
| *w; UAS-geminin^RNAi^* | geminin^KD^ | BDSC 30929 and 50720 |
| *w^1118^;GUS-p53* | UAS-p53^WT^ | BDSC 6584 |
| *y,w^1118^; UAS-p53 259N* | UAS-p53^DN^ | BDSC 6582 |
