# Supplementary material for: Polyploidy in the adult Drosophila brain
Source: eLife. 2020 Aug 25;9:e54385. doi: 10.7554/eLife.54385 (PMC7447450; doi:10.7554/eLife.54385)
Supplement: Supplementary file 3. [file elife-54385-supp3.docx]

**Supplemental Table. 3 Proportions of cell types polyploid in the whole brain**

| **Cell Type** | **% of total cells** | **% polyploid** | **% of total polyploidy** |
| --- | --- | --- | --- |
| All Neurons | 91% | 5.8% | 84.7% |
| All Glia | 8% | 6.3% | 13.2% |
| Tracheal cells | 0.3% | 100% | 2.8% |
| Sub Perineurial Glia | 1% | 100% | 4.5% |
