# Supplementary material for: Polyploidy in the adult Drosophila brain
Source: eLife. 2020 Aug 25;9:e54385. doi: 10.7554/eLife.54385 (PMC7447450; doi:10.7554/eLife.54385)
Supplement: Supplementary file 4. [file elife-54385-supp4.docx]

**Supplemental Table. 4 Proportions of polyploid cell types in the OL**

| **Cell Type** | **% of total cells** | **% polyploid** | **% of total polyploidy** |
| --- | --- | --- | --- |
| All neurons | 86% | 15%% | 85% |
| GABAergic neurons | 58% | 23% | 54% |
| Glutamatergic neurons | 35% | 22.6% | 33% |
| Cholinergic neurons | 25% | 11.7% | 15% |
| TM3a | 3.2% | 22.3% | 3.7% |
| DM9 | 1.3% | 23.6% | 2.2% |
| DM4 | 1.3% | 33.33% | 0.7% |
| DM10 | 5% | 33.1% | 2.8% |
| DM2 | 1.9% | 27% | 1.6% |
| All glia | 7% | 7.5% | 13% |
| Astrocyte-like glia | 2% | 17% | 1.1% |
| Cortex Glia | 5.6% | 28% | 7% |
| Wrapping glia | 3% | 35% | 5% |
